# Metabolic equivalents intensity thresholds for physical activity classification in older adults

**DOI:** 10.1186/s11556-024-00348-5

**Published:** 2024-05-21

**Authors:** Javier Leal-Martín, Miguel Muñoz-Muñoz, Miguel Sierra-Ramón, Mónica Cerezo-Arroyo, Paola Gómez-Redondo, Luis M. Alegre, Ignacio Ara, Francisco José García-García, Asier Mañas

**Affiliations:** 1 GENUD Toledo Research Group, Faculty of Sports Sciences, Universidad de Castilla- La Mancha, Av. Carlos III, 45071 Toledo, S/N Spain; 2 Faculty of Sports Sciences, Universidad de Castilla-La Mancha, Toledo, Spain; 3 CIBER On Frailty and Healthy Ageing (CIBERFES), Instituto de Salud Carlos III, Madrid, Spain; 4 Instituto de Investigación Sanitaria de Castilla- La Mancha (IDISCAM), Junta de Comunidades de Castilla- La Mancha (JCCM), Toledo, Spain; 5 Geriatric Department, Hospital Virgen del Valle, Complejo Hospitalario Universitario de Toledo, Toledo, Spain; 6 Center UCM- ISCIII for Human Evolution and Behavior, Madrid, Spain; 7 Faculty of Education, Complutense University of Madrid, Madrid, Spain

**Keywords:** Exercise, Metabolic equivalents, Energy expenditure, Older population, Cut-off points

## Abstract

**Background:**

Although the metabolic equivalents (METs) system is a common procedure to quantify the intensity of physical activity in older adults, it remains unclear whether the conventional METs intensity thresholds (CTs) used for this purpose are appropriate in this population. Therefore, this study aimed (i) to derive overall and fitness-specific METs intensity thresholds in older adults ≥ 60 years old (OATs) expressed both in standard METs (VO_2_/3.5 mL O_2_·kg^−1^·min^−1^) and older adults METs_60+_ (VO_2_/2.7 mL O_2_·kg^−1^·min^−1^), and (ii) to compare them with the CTs.

**Methods:**

A total of 93 subjects were assessed for cardiorespiratory fitness. Graded exercise test protocols using indirect calorimetry were performed to calculate individual VO_2max_ and categorize subjects as "very poor/fair" or "good/superior" fitness. Overall and fitness-specific OATs expressed in standard METs (OATs_standard_) and METs_60+_ (OATs_60+_) were derived based on the %VO_2max_ and the ventilatory thresholds (VTs) physical intensity categories.

**Results:**

Significantly higher VO_2max_, VO_2_ at VT_1_ and VO_2_ at VT_2_ (*p* < 0.001) were obtained in the "good/superior" subgroup compared to the "very poor/fair" fitness subgroup. Accordingly, OATs were approximately 69% higher in individuals with a "good/superior" fitness compared to those with a "very poor/fair" fitness. Furthermore, this study showed that OATs_standard_ were approximately 21–24% lower than OATs_60+_, and 10–22% higher OATs were observed when following the VTs intensity categories (heavy-intensity physical activity [HPA] and severe-intensity physical activity [SPA]) compared to the %VO_2max_ categories (moderate-intensity physical activity [MPA] and vigorous-intensity physical activity [VPA]). When compared with the CTs, similar or higher OATs_standard_ and OATs_60+_ for MPA, and HPA were obtained compared to the conventional MPA threshold (3.0 METs). Conversely, for VPA and SPA, lower, similar, or higher OATs were obtained depending on the METs derivation approach (OATs_standard_ or OATs_60+_) or the intensity categories (VO_2max_ or VTs), compared to the conventional VPA threshold (6.0 METs).

**Conclusions:**

None of the derived OATs were concurrently similar to the CTs, suggesting that fitness-specific METs intensity thresholds adapted to the METs derivation approach should be used in older adults.

**Trial registration:**

FenotipAGING (Non-health-care intervention study), PRO-Training (NCT05619250).

**Supplementary Information:**

The online version contains supplementary material available at 10.1186/s11556-024-00348-5.

## Introduction

Metabolic equivalents (METs) are a physiological concept widely used in epidemiology to quantify the absolute intensity of physical activity using accelerometer devices, questionnaires, and diary/logs [1, 2]. METs are expressed as multiples of a standardized resting metabolic rate (RMR) value of 3.5 mL O_2_·kg^−1^·min^−1^ [3, 4]. These standard METs (VO_2_/3.5 mL O_2_·kg^−1^·min^−1^) are used with conventional METs intensity thresholds (CTs) that, as defined in the 2018 Physical Activity Guidelines Advisory Committee Scientific Report [5], classify exertion as light-intensity physical activity (LPA, > 1.5 to < 3.0 METs), moderate-intensity physical activity (MPA, ≥ 3.0 to < 6.0 METs) or vigorous-intensity physical activity (VPA, ≥ 6.0 METs) [5].

Despite the widespread use of the METs system, several studies have suggested that using standard METs may misrepresent physical intensity in the older adult population [6, 7]. Evidence points to several age-related factors, such as body composition and clinical status, that may condition its use [1, 8–10]. In this regard, a systematic review by Leal-Martín [11] reported a weighted average RMR value of 2.7 mL O_2_·kg^−1^·min^−1^ in older adults ≥ 60 years old, which is 23% lower than the standard value of 1 MET [11]. In addition, given that cardiorespiratory fitness declines with age [12], it is still unclear whether the CTs application may also contribute to misclassify the intensity of physical activity in this population. As proposed by Willis [13] in the 2024 Older Adult Compendium of Physical Activities, additional research is also needed to examine the use of the CTs when combined with alternative METs derivation approaches such as multiples of the aforementioned older adult-based RMR value of 2.7 mL O_2_·kg^−1^·min^−1^ (VO_2_/2.7 mL O_2_·kg^−1^·min^−1^), also referred as METs_60+_ [11, 13]. Moreover, the American College of Sports Medicine (ACSM) [2] has previously reported METs intensity thresholds for the older population (ACSMTs). These ACSMTs are expressed in standard METs, categorizing the physical intensity according to the percentage of a maximal aerobic capacity (%VO_2max_) of 8 METs [2, 14, 15]. However, these intensity thresholds are not without limitations. First, they may not be applied when using METs_60+_. Second, the methodological procedures followed to obtain the VO_2max_ are vaguely described. Third, these METs intensity thresholds assume a fixed reference VO_2max_ that may not be suitable for both lower or higher fitness individuals [16, 17]. Finally, the ACSMTs are based on the %VO_2max_ intensity categories paradigm, which have been criticized for not adequately controlling the metabolic stimulus [18, 19]. Instead, the use of intensity categories based on ventilatory thresholds (VTs) is recommended as they correspond to more meaningful physiological events during physical activity (metabolic pathways, energy substrates, accumulation of lactate and metabolites in the blood, etc.) and fatigue. This would allow for better comparability between studies, leading to improved accuracy in both the physical activity dose–response relationship with health outcomes, and health-related physical activity guidelines from a clinical and epidemiological perspective.

Based on the above, it is necessary to derive alternative METs intensity thresholds in older adults ≥ 60 years old (OATs) expressed both in standard METs (OATs_standard_) and METs_60+_ (OATs_60+_) to understand the actual misclassification that occurs when CTs are used in the older population. In this regard, several hypotheses can be formulated: First, it is expected that OATs_standard_, following the VO_2max_ intensity categories, will be lower than the CTs for both MPA and VPA. Second, OATs_standard_, following the VO_2max_ intensity categories, will be closer to the CTs in individuals with higher fitness level. Third, higher OATs will be observed when expressed in METs_60+_ or when following the VTs intensity categories. Therefore, the main aims of this study were (i) to derive overall and fitness-specific OATs_standard_ and OATs_60+_ obtained from graded exercise test (GXT) protocols, according to the %VO_2max_ and VTs physical intensity categories, and (ii) to compare them with the CTs of the METs system.

## Methods

### Participants

This work included a total of 93 adults ≥ 60 years old (46 women and 47 men). Participants belonged to two different studies conducted within the Growth, Exercise, Nutrition, and Development (GENUD) Toledo research group (UCLM, Toledo, Spain): (i) FenotipAGING study (*n* = 42; 69.5 ± 3.6 years) and (ii) Promoting Training Programmes for Health (PRO-Training) study (*n* = 51; 68.6 ± 4.2 years). The participating criteria were to be ≥ 65 years old (FenotipAGING) or ≥ 60 years old (PRO-Training). Older adults unable to walk independently, acute joint injury, or medical contraindication for exercise were excluded. All subjects gave their written informed consent before inclusion, and the study procedures were performed following the Declaration of Helsinki. All studies were approved by the Toledo Hospital Complex Ethics Committee in Toledo, Spain.

### Experimental design

The FenotipAGING and PRO-Training studies were conducted at the GENUD Toledo research group facilities, where all participants underwent a GXT to determine their VO_2max_. Participants from both studies were requested to attend all tests in a post-absorptive and euhydrated state, without consumption of any stimulant substance (e.g., caffeine, nicotine) for 4 h, and refraining from moderate or vigorous physical activity for 24 h and 48 h, respectively [20]. Subsequently, individuals were classified as "very poor/fair" or "good/superior" fitness, using specific sex and age thresholds from the "ACSM’s Health-related Physical Fitness Assessment Manual" [17] ([men, 60–69 years] "very poor/fair" ≤ 34.9 mL O_2_·kg^−1^·min^−1^, "good/superior" > 34.9 mL O_2_·kg^−1^·min^−1^, [men, 70–79 years] "very poor/fair" ≤ 31.5 mL O_2_·kg^−1^·min^−1^, "good/superior" > 31.5 mL O_2_·kg^−1^·min^−1^, [women, 60–69 years] "very poor/fair" ≤ 29.4 mL O_2_·kg^−1^·min^−1^, "good/superior" > 29.4 mL O_2_·kg^−1^·min^−1^, [women, 70–79 years] "very poor/fair" ≤ 28.0 mL O_2_·kg^−1^·min^−1^, "good/superior" > 28.0 mL O_2_·kg^−1^·min^−1^).

### GXT and verification test

Participants from the FenotipAGING and PRO-Training studies underwent a GXT and a supramaximal constant load verification test (VerT) on an electromagnetically braked cycle-ergometer (800S, Ergoline, Bitz, Germany). O_2_ consumed and CO_2_ produced were assessed by indirect calorimetry (FenotipAGING: Oxycon Pro, Erich Jaeger GmbH, Hoechberg, Germany; PRO-Training: Cosmed Quark RMR, Cosmed srl, Rome, Italy) using breath-by-breath mode. Metabolic devices were previously calibrated according to the manufacturer's instructions [21, 22]. Heart rate (HR) was also continuously recorded and synchronized with the software of the metabolic device using a standard 12-lead electrocardiogram (Cardiosoft 12SL-ECG, GE Healthcare, Finland). For the GXTs, the PRO-Training study followed a sex-specific protocol, and the FenotipAGING performed a protocol according to self-reported physical activity status with sex-specific variants. In broad terms, both GXT protocols had an initial warm-up phase followed by a loading phase with an active/passive recovery period after completion. After recovery, a VerT was performed, challenging participants to exert themselves to 110% W_max_ achieved during the GXT. During the incremental and supramaximal load protocols, individuals were required to pedal at a constant cadence between 60 and 90 rpm, being verbally encouraged until volitional cessation. Detailed information on the GXT protocols can be found in Supplementary Table 1 and 2.

### Complementary descriptive outcomes

Additional outcomes including RMR estimates, and physical performance status were calculated. The RMR (mL O_2_·kg^−1^·min^−1^) was obtained using the predictive equations derived by Byrne [1] (RMR_Byrne_: 3.6145—0.0367 [Body mass index (BMI)]—0.0038 [age] + 0.1790 [gender]), Lührmann [23] (RMR_Lührmann_: 3169 + 50.0 [weight] − 15.3 [age] + 746 [sex]), and Harris [24] (RMR_Harris- Benedict_: men: 655.0955 + 9.5634 [weight] + 1.8496 [height] − 4.6756 [age]; women: 66.4730 + 13.7516 [weight] + 5.0033 [height] − 6.7550 [age]) as a descriptive estimation of the measured RMR value in the study participants. Finally, the physical performance of all participants in both studies was assessed using the Short Physical Performance Battery (SPPB) [25].

### Data processing

Metabolic and ventilatory data from the GXT and VerT was averaged over 20 s. The GXT was assumed to meet the plateau criteria when the increase in VO_2_ between the next-to-last period and the maximal work rate achieved in the GXT was ≤ 50% of the expected for that increase in the work rate [26]. Similarly, the GXT was verified when the increase in VO_2_ between the maximum work rate in the GXT and the supramaximal work rate in the VerT (110% W_max_) was ≤ 50% of that expected [27]. The maximum VO_2_ values achieved during the GXT and VerT were derived from the period with the highest O_2_ consumption in which the individual maintained a pedaling cadence of at least 60 rpm. VO_2max_ was selected to be the highest value achieved between the two protocols. Thereafter, VTs in the GXT were visually derived for each subject. For the ventilatory threshold 1 (VT_1_) the Beaver's V-slope method was taken as a reference although cross-checked using the O_2_ ventilatory equivalent method and the end-tidal O_2_ pressure method [28, 29]. As for the ventilatory threshold 2 (VT_2_), the CO_2_ ventilatory equivalent method (VE/VCO_2_) was set as a reference, but also cross-checked using the end-tidal CO_2_ pressure method. Those VTs were determined according to the best agreement between two independent observers (JLM and MS) and disagreement was resolved with a third evaluator (MM). Finally, VO_2_ measures were converted to METs using two different derivation approaches: standard METs (VO_2_/3.5 mL O_2_·kg^−1^·min^−1^) [3] and METs_60+_ (VO_2_/2.7 mL O_2_·kg^−1^·min^−1^) [11, 13].

### Statistical analyses

Descriptive data are presented as mean and standard deviation (SD) or frequency (n) and percentage (%) for the overall sample and by fitness subgroups. Additionally, these data are also graphically displayed, by fitness subgroup, using raincloud plots (data distribution plot, box plot and raw data). Differences in the descriptive data between fitness subgroups were tested using unpaired sample Student’s t-tests with a confidence interval of 95% and an *α* = 0.05. Overall and fitness-specific OATs_standard_ and OATS_60+_ were calculated according to the physical intensity categories from the %VO_2max_ and the VTs. Regarding the %VO_2max_ categories, OATs for MPA and VPA were determined at 46% and 64% of individual VO_2max_, respectively [20]. For the VTs categories, OATs for heavy-intensity physical activity (HPA) and severe-intensity physical activity (SPA) were respectively determined at individual VT_1_ and VT_2_. OATs for the overall sample and by fitness subgroups ("very poor/regular" and "good/superior") [17] were set at the mean VO_2_ in each physiological event (46%VO_2max_, 64%VO_2max_, VT_1_, VT_2_). The obtained overall and fitness-specific OATs_standard_ and OATS_60+_ were visually compared with the CTs. Finally, post-hoc power computation analyses, using the G*Power 3.1 software [30], were performed on the primary outcomes of VO_2max_ (mL O_2_·kg^−1^-min^−1^), VT_1_ (mL O_2_·kg^−1^·min^−1^), and VT_2_ (mL O_2_·kg^−1^·min^−1^). Based on the fitness-specific means, an effect size of 0.8 (large) was set, assuming an *α* = 0.05, with a sample size of 71 subjects in the "very poor/fair" fitness subgroup and 22 subjects in the "good/superior" fitness subgroup.

## Results

Descriptive data for the overall sample and by fitness subgroup is presented in Table 1, while raincloud plots provide a visual representation of the data by fitness subgroup (Supplementary Figs. 1, 2, 3, 4 and 5). Post-hoc computation analyses achieved a statistical power (1-*β* probability error) above 0.90 in all tested primary outcomes. Significantly higher values for VO_2max_, work rate, and HR at VO_2max_, and VO_2_ at VTs were observed in the “good/superior” fitness subgroup. Conversely, no differences were observed regarding respiratory exchange ratio (RER), and significantly lower estimated RMR was observed in the “very poor/fair” fitness subgroup, except in the RMR_Lührmann_. Differences in sex, height, and BMI were found, and all study participants were classified as robust on the SPPB.

**Table 1:** Sample characteristics

	Overall sample (*n* = 93)	Very poor/fair fitness subgroup (*n* = 71)	Good/superior fitness subgroup (*n* = 22)	*p*-value
Sex (n)^a^				
Men	47 (50.5)	28 (39.4)	19 (86.4)	** < 0.001**
Women	46 (49.5)	43 (60.6)	3 (13.6)	
Age (years)^b^	69.0 (3.9)	68.7 (4.0)	70.1 (3.6)	0.143
Weight (kg)^b^	70.3 (12.0)	71.2 (12.9)	67.4 (7.8)	0.104
Height (cm)^b^	164.2 (7.9)	163.0 (7.6)	168.1 (7.6)	**0.007**
BMI (kg·m^−2^)^b^	26.0 (3.6)	26.7 (3.7)	23.8 (2.2)	** < 0.001**
SPPB (score)	11.8 (1.3)	11.8 (1.5)	12.0 (0.0)	0.445
RMR				
RMR_Byrne_ (mL O_2_·kg^−1^·min^−1^)^b,c^	2.7 (0.1)	2.6 (0.1)	2.8 (0.1)	** < 0.001**
RMR_Lührmann_ (mL O_2_·kg^−1^·min^−1^)^b,d^	3.0 (0.4)	3.0 (0.4)	2.9 (0.3)	0.053
RMR_Harris- Benedict_ (mL O_2_·kg^−1^·min^−1^)^b,e^	2.8 (0.2)	2.8 (0.2)	2.9 (0.1)	**0.006**
VO_2max_				
Source (n)^a^				
GXT	36 (39)	27 (38)	9 (41)	0.811
VerT	57 (61)	44 (62)	13 (59)	
VO_2_ (mL O_2_·kg^−1^·min^−1^)^b^	27.8 (8.4)	23.9 (4.2)	40.5 (5.4)	** < 0.001**
VO_2_ (mL O_2_·min^−1^)^b^	1952.3 (646.2)	1707.5 (459.5)	2742.1 (519.3)	** < 0.001**
RER^b^	1.1 (0.1)	1.1 (0.1)	1.1 (0.1)	0.271
HR (bpm)^b^	143.3 (17.4)	141.5 (18.6)	148.9 (11.2)	**0.028**
Work rate (W)^b^	125.7 (56.7)	103.1 (38.1)	198.6 (44.1)	** < 0.001**
Standard METs^b,f^	8.0 (2.4)	6.8 (1.2)	11.6 (1.5)	** < 0.001**
METs_60+_^b,g^	10.3 (3.1)	8.9 (1.6)	15.0 (2.0)	** < 0.001**
VT_1_				
VO_2_ (mL O_2_·kg^−1^·min^−1^)^b^	14.7 (4.6)	12.8 (2.9)	20.6 (4.3)	** < 0.001**
VO_2_ (mL O_2_·min^−1^)^b^	1021.0 (333.6)	906.5 (239.5)	1390.5 (329.9)	** < 0.001**
RER	0.9 (0.1)	0.9 (0.1)	0.9 (0.1)	0.822
VT_2_				
VO_2_ (mL O_2_·kg^−1^·min^−1^)^b^	21.2 (7.2)	18.0 (3.7)	31.3 (6.3)	** < 0.001**
VO_2_ (mL O_2_·min^−1^)^b^	1491.7 (536.0)	1290.4 (357.1)	2123.0 (517.4)	** < 0.001**
RER	1.0 (0.1)	1.0 (0.1)	1.0 (0.1)	0.397

*BMI* body mass index, *GXT* graded exercise test, *HR* heart rate, *RER* respiratory exchange ratio, *RMR* resting metabolic rate, *SD* standard deviation, *SPPB* short physical performance battery, *VCO*_2_ carbon dioxide production, *VerT* supramaximal verification test, *VO*_2_ oxygen uptake, *VT*_1_ ventilatory threshold 1, *VT*_2_ ventilatory threshold 2

^a^Categorical variable: n (%)

^b^Continuous variable: mean (SD)

^c^RMR derived using the Byrne equation[1]

^d^RMR derived using the Lührmann equation[23]

^e^RMR derived using the Harris-Benedict equation[24]

^f^VO_2max_/3.5 mL O_2_·kg^−1^·min^−1^

^g^VO_2max_/2.7 mL O_2_·kg^−1^·min^−1^

Bold: Statistical significance at *p* ≤ 0.050

**Fig. 1:**
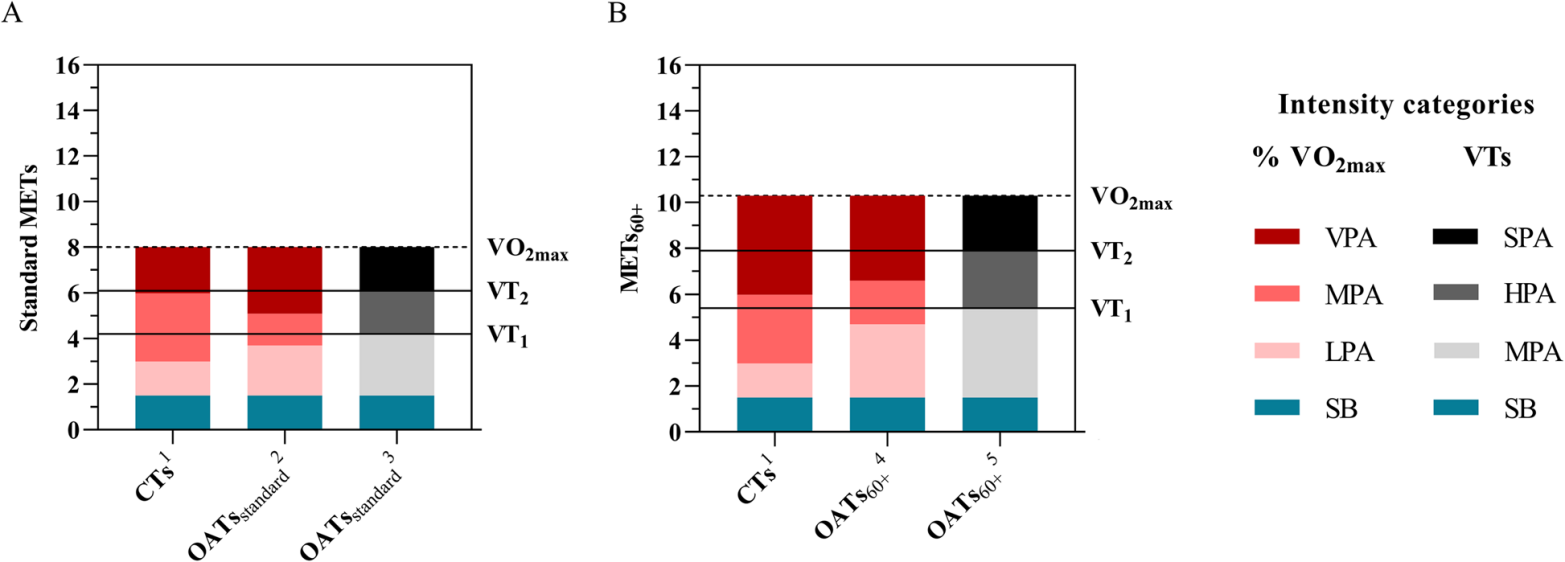
Overall OATs ranges compared to the CTs ranges. CTs: conventional METs intensity thresholds, HPA: heavy-intensity physical activity, LPA: light-intensity physical activity, METs: metabolic equivalents, MPA: moderate-intensity physical activity, OATs: METs intensity thresholds for older adults ≥ 60 years old, OATs_standard_: OATs expressed in standard METs (VO_2_/3.5 mL O_2_·kg^−1^·min^−1^), OATs_60+_: OATs expressed in METs_60+_ (VO_2_/2.7 mL O_2_·kg^−1^·min^−1^), SB: sedentary behaviour, SPA: severe-intensity physical activity, VO_2max_: maximal aerobic capacity, VPA: vigorous-intensity physical activity, VTs: ventilatory thresholds, VT_1_: ventilatory threshold 1, VT_2_: ventilatory threshold 2. ^1^[SB] ≤ 1.5 METs, [LPA] > 1.5 to < 3.0 METs, [MPA] ≥ 3.0 to < 6.0 METs, [VPA] ≥ 6.0 METs; ^2^[SB] ≤ 1.5 METs, [LPA] > 1.5 to < 3.7 METs, [MPA] ≥ 3.7 to < 5.1 METs, [VPA] ≥ 5.1 METs; ^3^[SB] ≤ 1.5 METs; [MPA] > 1.5 to < 4.2 METs, [HPA] ≥ 4.2 to < 6.1 METs, [SPA] ≥ 6.1 METs; ^4^[SB] ≤ 1.5 METs, [LPA] > 1.5 to < 4.7 METs, [MPA] ≥ 4.7 to < 6.6 METs, [VPA] ≥ 6.6 METs; ^5^[SB] ≤ 1.5 METs, [MPA] > 1.5 to < 5.4 METs, [HPA] ≥ 5.4 to < 7.9 METs, [SPA] ≥ 7.9 METs

**Fig. 2:**
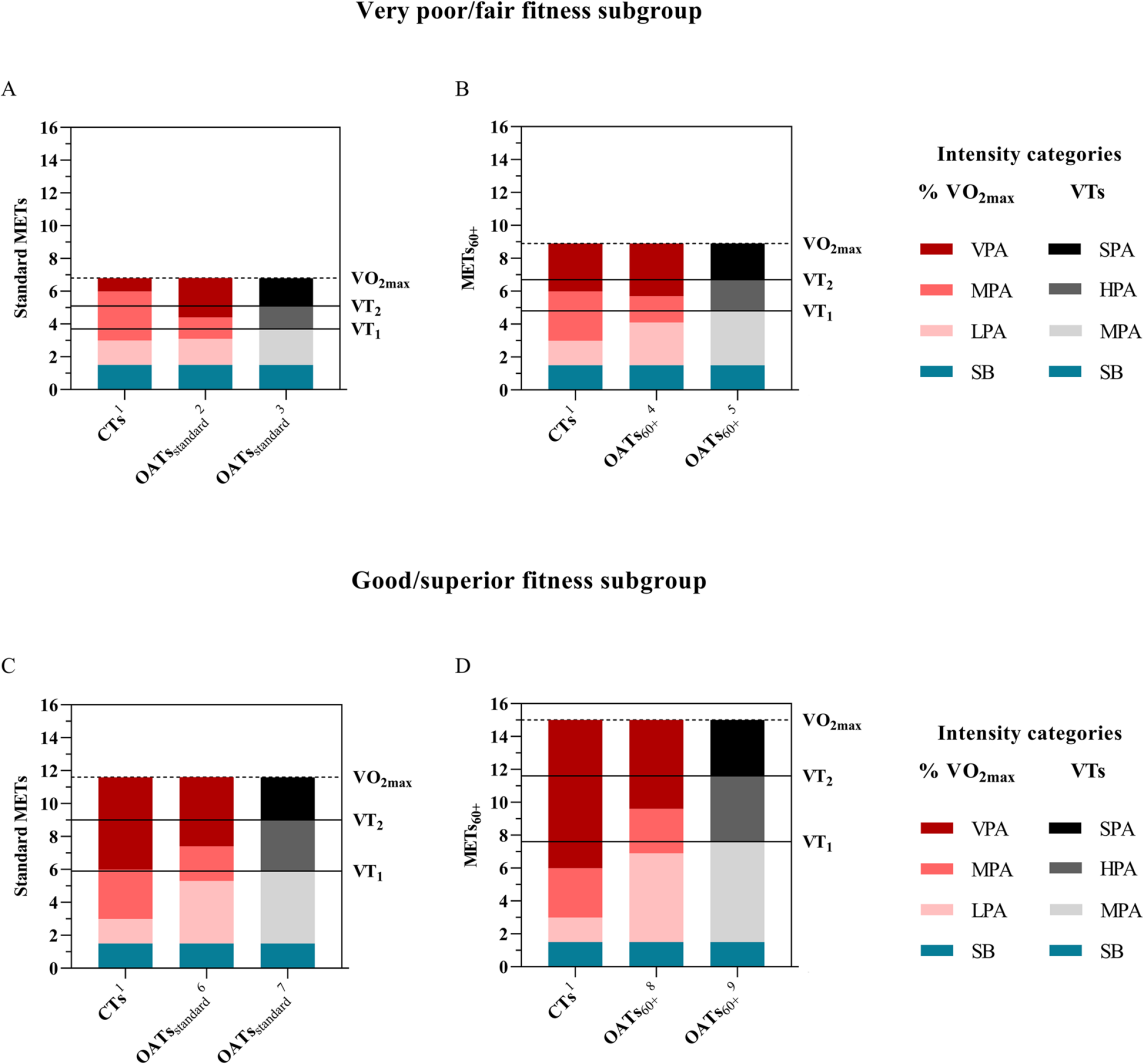
Fitness-specific OATs ranges compared to the CTs ranges. CTs: conventional METs intensity thresholds, HPA: heavy-intensity physical activity, LPA: light-intensity physical activity, METs: metabolic equivalents, MPA: moderate-intensity physical activity, OATs: METs intensity thresholds for older adults ≥ 60 years old, OATs_standard_: OATs expressed in standard METs (VO_2_/3.5 mL O_2_·kg^−1^·min^−1^), OATs_60+_: OATs expressed in METs_60+_ (VO_2_/2.7 mL O_2_·kg^−1^·min^−1^), SB: sedentary behaviour, SPA: severe-intensity physical activity, VO_2max_: maximal aerobic capacity, VPA: vigorous-intensity physical activity, VTs: ventilatory thresholds, VT_1_: ventilatory threshold 1, VT_2_: ventilatory threshold 2. ^1^[SB] ≤ 1.5 METs; [LPA] > 1.5 to < 3.0 METs; [MPA] ≥ 3.0 to < 6.0 METs; [VPA] ≥ 6.0 METs, ^2^[SB] ≤ 1.5 METs; [LPA] > 1.5 to < 3.1 METs; [MPA] ≥ 3.1 to < 4.4 METs; [VPA] ≥ 4.4 METs, ^3^[SB] ≤ 1.5 METs; [MPA] > 1.5 to < 3.7 METs; [HPA] ≥ 3.7 to < 5.1 METs; [SPA] ≥ 5.1 METs, ^4^[SB] ≤ 1.5 METs; [LPA] > 1.5 to < 4.1 METs; [MPA] ≥ 4.1 to < 5.7 METs; [VPA] ≥ 5.7 METs, ^5^[SB] ≤ 1.5 METs; [MPA] > 1.5 to < 4.8 METs; [HPA] ≥ 4.8 to < 6.7 METs; [SPA] ≥ 6.7 METs, ^6^[SB] ≤ 1.5 METs; [LPA] > 1.5 to < 5.3 METs; [MPA] ≥ 5.3 to < 7.4 METs; [VPA] ≥ 7.4 METs, ^7^[SB] ≤ 1.5 METs; [MPA] > 1.5 to < 5.9 METs; [HPA] ≥ 5.9 to < 9.0 METs; [SPA] ≥ 9.0 METs, ^8^[SB] ≤ 1.5 METs; [LPA] > 1.5 to < 6.9 METs; [MPA] ≥ 6.9 to < 9.6 METs; [VPA] ≥ 9.6 METs, ^9^[SB] ≤ 1.5 METs; [MPA] > 1.5 to < 7.6 METs; [HPA] ≥ 7.6 to < 11.6 METs; [SPA] ≥ 11.6 METs

Disparate overall (Fig. 1) and fitness-specific (Fig. 2) OATs derived from this sample of older adults were observed. On the one hand, OATs varied depending on the METs derivation approach, with OATs_standard_ being 21–24% lower than OATs_60+_. Moreover, fitness-specific OATs derived from "very poor/fair" and “good/superior” fitness individuals showed 11–16% lower, and 40–48% higher thresholds compared to the overall OATs (Table 2), respectively. Furthermore, higher OATs were reported when based on the VTs intensity categories compared to those based on the %VO_2max_ intensity categories, with 10–19% higher HPA than MPA thresholds and 16–22% higher SPA than VPA thresholds.

**Table 2:** METs intensity thresholds for older adults ≥ 60 years old

			Overall (*n* = 93)	Very poor/fair fitness subgroup (*n* = 71)	Good/superior fitness subgroup (*n* = 22)
	VO_2_ event	Intensity category	Threshold^c^	Threshold^c^	Threshold^c^
OATs_standard_^a^	46% VO_2max_	MPA	3.7 (1.1)	3.1 (0.6)	5.3 (0.7)
	64% VO_2max_	VPA	5.1 (1.5)	4.4 (0.8)	7.4 (1.0)
	VT_1_	HPA	4.2 (1.3)	3.7 (0.8)	5.9 (1.2)
	VT_2_	SPA	6.1 (2.1)	5.1 (1.0)	9.0 (1.8)
OATs_60+_^b^	46% VO_2max_	MPA	4.7 (1.4)	4.1 (0.7)	6.9 (0.9)
	64% VO_2max_	VPA	6.6 (2.0)	5.7 (1.0)	9.6 (1.3)
	VT_1_	HPA	5.4 (1.7)	4.8 (1.1)	7.6 (1.6)
	VT_2_	SPA	7.9 (2.7)	6.7 (1.4)	11.6 (2.3)

*HPA* heavy-intensity physical activity, *METs* metabolic equivalents, *MPA* moderate-intensity physical activity, *OATs* METs intensity thresholds for older adults ≥ 60 years old, *SD* standard deviation, *SPA* severe-intensity physical activity, *VO*_2_ oxygen uptake, *VO*_2*max*_ maximal oxygen uptake, *VPA* vigorous-intensity physical activity, *VT*_1_ ventilatory threshold 1, *VT*_2_ ventilatory threshold 2

^a^OATs expressed in standard METs (VO_2_/3.5 mL O_2_·kg^−1^·min^−1^)

^b^OATs expressed in METs_60+_ (VO_2_/.2.7 mL O_2_·kg^−1^·min^−1^)

^c^Mean (SD)

On the other hand, none of the derived OATs were concurrently similar to those from the CTs. When compared with the conventional MPA threshold (3.0 METs), only the OATs_standard_, following the %VO_2max_ intensity categories, in the “very poor/fair” fitness subgroup showed a similar value (3.1 METs, Fig. 2A). In the remaining OATs_standard_ and OATs_60+_, higher MPA and HPA thresholds were obtained. Therefore, using the conventional MPA threshold would overestimate physical intensity in this sample of older adults, except in the poorer fitness older individuals. However, disparate findings were found when the derived VPA and SPA thresholds were compared with the conventional VPA threshold (6.0 METs). Therefore, according to the METs derivation approach (OATs_standard_ or OATs_60+_), or the fitness subgroup (“very poor/fair” or “good/superior”), lower, similar, or higher OATs were obtained. For example, the OATs_standard_ following the %VO_2max_ intensity categories showed lower VPA thresholds both in the overall sample (5.1 METs, Fig. 1A) and the “very poor/fair” fitness subgroup (4.4 METs, Fig. 2A) but higher in the “good/superior” fitness subgroup (7.4 METs, Fig. 2C) compared with the conventional one (6 METs). However, these VPA thresholds had an opposite sense when expressed in METs_60+_ (OATs_60+_), reporting higher thresholds in the overall sample (6.6 METs, Fig. 1B), and similar in the “very poor/fair” fitness subgroup (5.7 METs, Fig. 2B). Therefore, the conventional VPA threshold would underestimate or overestimate physical intensity according to the fitness subgroup, and the METs derivation approach used.

## Discussion

To our knowledge, this is the first study to derive overall and fitness-specific OATs_standard_ and OATs_60+_, following the %VO_2max_ and the VTs physical intensity categories, and to compare them with the CTs. Briefly, this work highlights the importance of using METs intensity thresholds adapted to both the assumed 1 MET value and the fitness status of older individuals. In this regard, OATs_standard_ were found to be 21–24% lower compared to OATs_60+_, and 58–76% higher in "good/superior" fitness individuals than in "very poor/fair" fitness individuals. When compared with the CTs, higher OATs_standard_ and OATs_60+_ were obtained for MPA and HPA than the conventional MPA threshold (3.0 METs), except in the “very poor/fair” fitness individuals. However, lower, similar, or even higher OATs_standard_ and OATs_60+_ were obtained for VPA, and SPA compared to the conventional VPA threshold (6.0 METs). As a result, this study revealed appreciable differences between the CTs and the derived OATs, even when calculated following the CTs paradigm (standard METs and %VO_2max_ intensity categories). Therefore, a potential for CTs to misclassify LPA, MPA and VPA in older adults can be inferred, primarily depending on the cardiorespiratory fitness and the METs derivation approach. Alternatively, this study provides resources for the adaptation of the METs system in this population, reporting OATs_standard_ and OATs_60+_ according to the fitness status, and following habitual (%VO_2max_) and alternative (VTs) physical intensity categories.

### Comparing the CTs with the overall and fitness-specific OATs

Looking at the OATs_standard_ following the VO_2max_ intensity categories, equal or higher MPA thresholds and lower, similar, or higher VPA thresholds were reported according to the fitness subgroup, compared to those of the CTs (Fig. 1A, Fig. 2A). These results partially refute the first proposed hypotheses that both the MPA and VPA thresholds would be lower than those from the CTs, suggesting that the main factor in the correct categorization of physical intensity lies in the level of fitness. In this sense, the derived OATs_standard_ for MPA were higher in the overall sample (3.7 METs, Fig. 1A) and the “good/superior” fitness subgroup (5.3 METs, Fig. 2C), but nearly identical in the “very poor/fair” fitness individuals (3.1 METs, Fig. 2A) compared to the conventional one (3.0 METs). On the other hand, the OATs_standard_ for VPA were substantially higher in the “good/superior” fitness subgroup (7.4 METs, Fig. 2A), but notably lower in the overall sample (5.1 METs, Fig. 1A) and the “very poor/fair” fitness subgroup (4.4 METs, Fig. 2A). Indeed, older adults in this “very poor/fair” fitness subgroup would need to exceed 88% of their VO_2max_ to achieve the 6.0 METs of the conventional VPA threshold, which is 13% higher than the VT_2_ (SPA: 5.1 METs, Fig. 2A) in this subgroup. This contrast with the usual assumption that older adults are physiologically less able to achieve absolute VPA, and explains why moderate-to-vigorous physical activity is often used as a pooled domain of intense physical activity [31]. Similar METs intensity thresholds to those from the "very poor/fair" fitness subgroup (OATs_standard_, %VO_2max_, MPA: 3.1 METs, VPA: 4.4 METs) has been previously reported by the ACSM, suggesting ACSMTs of 3.2 and 4.8 METs for MPA and VPA, respectively (Supplementary Fig. 1). In contrast, 68–71% higher OATs_standard_ following the VO_2max_ physical activity intensity categories, for MPA (5.3 METs, Fig. 2C) and VPA (7.4 METs, Fig. 2C) were respectively derived in the "good/superior" fitness individuals, compared to those in the “very poor/fair” fitness subgroup (MPA: 3.1 METs, VPA: 4.4 METs, Fig. 2A). This difference was notably greater that that shown by Mendes [32] in a sample from 20 to 60 years old, obtaining 36% and 22% higher MPA and VPA thresholds in the high fitness compared to the low fitness individuals, respectively. These results directly refute the second hypothesis of the present study, which expected similar OATs_standard_, following the %VO_2max_ physical intensity categories, in the higher fitness subgroup compared to the CTs. Therefore, although older age is associated with lower cardiorespiratory fitness [12, 33], a homogeneous criterion should not be followed, and the use of non-fitness-specific METs intensity thresholds may increase the risk of inaccurately classifying physical activity intensity. Furthermore, based on the disparate prevalence of sedentary and inactive lifestyle in older adults [34], caution should be taken if the CTs are used in this population.

### Comparing the CTs with the OATs_standard_ and the OATs_60+_

As for the METs derivation approaches used, 21–24% lower OATs_standard_ compared to the OATs_60+_ were obtained. The reason for this difference is the 1 MET value 23% lower than the standard assumed when deriving the OATs_60+_. These results align with the third hypothesis, expecting higher OATs when using METs_60+_ than standard METs. Thus, since the CTs and the OATs_standard_ are based on standard METs, it is reasonable to consider that they should not be applied when assuming a 1 MET value far from 3.5 mL O_2_·kg^−1^·min^−1^. Therefore, the OATs_60+_ might be the preferred option when categorizing for the intensity of those METs_60+_ equivalencies from the Older Adult Compendium of Physical Activities, [13] but also when using estimated or measured RMR values close to 2.7 mL O_2_·kg^−1^·min^−1^. However, no study has compared the use of alternative METs system strategies, including variations in the 1 MET assumption or the METs intensity thresholds applied, with the “classical” one in older adults. Therefore, future studies should evaluate the differences between these two approaches, and whether significant improvements in the reliability of the conclusions are observed from an epidemiological and clinical perspective.

### Comparing the CTs with the OATs (VO_2max_) and the OATs (VTs)

This study also reported OATs following the VTs intensity categories (HPA and SPA), showing, as initially hypothesized, consistently higher METs intensity thresholds than the same OATs following the %VO_2max_ intensity categories (MPA and VPA). For instance, the overall sample’s OATs_standard_, following the VTs physical activity intensity categories, showed an HPA threshold of 4.2 METs (53%VO_2max_) and a SPA threshold of 6.1 METs (76%VO_2max_), which are respectively higher compared to those obtained at 46% (MPA: 3.7 METs, Fig. 1A) and 64%VO_2max_ (VPA: 5.1 METs, Fig. 1A) using the %VO_2max_ intensity categories. However, when these same overall OATs_standard_ were compared to the CTs, a similar SPA threshold (6.1 METs, 75% of VO_2max_, Fig. 1A) to the conventional VPA threshold (6.0 METs) was obtained. This issue was previously studied by Iannetta [16], who found a high risk of physical intensity misclassification when using "fixed universal METs" thresholds (LPA, MPA, VPA, maximal) compared to using VTs intensity categories (MPA, HPA, and SPA). These authors showed an increase in the HPA range as VO_2max_ increased, in contrast to the fixed MPA range in the CTs (≥ 3.0 METs to < 6.0 METs). Similar results were found in the present study, reporting HPA ranges in the OATs_standard_, following the VTs physical activity intensity categories, of 1.4 METs in the "very poor/fair" fitness subgroup and 3.1 METs in the "good/superior" fitness subgroup. Considering the importance of VTs from a physiological perspective, their use should be encouraged, providing more far-reaching evidence, including more certainty about metabolic pathways, the use of energy substrates, or metabolic stress during a given intensity category.

### Perspectives

This study suggests that a non-adapted use of the METs system in older adults may inadvertently undermine the validity of physical activity recommendations and prescription from both public health and clinical perspectives. Among the concerns this may entail, the unfair application of the conventional VPA threshold (6 METs) is one of the most significant, requiring older individuals with low fitness to achieve near-maximal effort (6.8 METs). This may explain the low levels of VPA frequently detected in older adults, many of whom lead sedentary and inactive lifestyles, and the widespread use of moderate-to-vigorous physical activity in this population [35]. Furthermore, if this low “absolute” VPA accumulation is then combined with MPA, the distinct effects these activity intensities may have separately is obscured, thereby limiting the depth of research findings. This poses new methodological challenges focused on the proper assessment of both fitness and physical activity in older adults. As there is now a global trend towards precision medicine, accurate estimation of physical activity intensity is essential [36, 37]. This aligns with recommendations for utilizing intensity categories based on the VTs, which better capture the metabolic stimulus and can improve comparability across studies. Otherwise, the validity of research delving into the dose amount and intensity of the physical activity needed to improve health will be compromised, reducing the efficacy, efficiency, and safety of physical activity interventions [38]. This is particularly critical for clinical subgroups, highlighting those with cardiovascular diseases [20, 39]. Therefore, a one-size-fits-all approach in the older population may not be appropriate, and personalized exercise programs considering individual fitness are essential for optimizing health outcomes. To this end, new approaches should also be explored by deriving individualized METs intensity thresholds based on additional determinants such as measured or estimated RMR, sex, body composition, physical performance, or clinical status [12, 33, 40].

This work presents various strengths and limitations. To our knowledge, this is the first study to provide overall and fitness-specific OATs_standard_ and OATs_60+_ based on GXT protocols in a relatively large older adult sample, and using two different approaches to categorize physical intensity. However, this study included data from two separate studies, FenotipAGING and PRO-Training. Since neither of them was originally designed to address our specific research question, we did not perform a priori power and sample size calculations. Therefore, we should acknowledge that these OATs might not apply satisfactorily in a different sample. Nevertheless, post-hoc power computation analyses were conducted afterwards, revealing a large effect size and achieving a statistical power (1-β probability error) above 0.90 for all primary outcomes. It should also be noted that, according to the methodologies in the FenotipAGING and the PRO-Training studies, subjects followed different GXT protocols, and distinct metabolic devices were used, which could limit to some extent the comparability of VO_2max_ among subjects. However, regarding the GXT protocols, no differences in RER were detected among subgroups, suggesting that cardiorespiratory fitness was consistently assessed across individuals. Furthermore, meticulous attention was paid when calibrating both metabolic devices, and standardizing the measurement conditions. Besides the OATs_standard_, this study also reported OATs_60+_ that could be useful to categorize the intensity of the METs_60+_ based equivalences from the Older Adult Compendium of Physical Activities, or when assuming a measured or estimated individual RMR closer to 2.7 mL O_2_·kg^−1^·min^−1^. Furthermore, OATs_standard_ and OATs_60+_ following two different strategies for physical activity intensity categorization were developed, highlighting the OATs based on VTs intensity categories. This will allow for more physiologically meaningful findings. Therefore, this study creates a new scenario by providing alternative strategies for adapting the METs system for improved use in adults ≥ 60 years old. Nevertheless, future studies should test whether using OATs, compared to the CTs, can make a real difference from a clinical and epidemiological perspective.

## Conclusion

The OATs derived from a sample of older adults ≥ 60 years old were notably different compared to those from the METs system. As a result, the CTs mostly underestimated the MPA threshold compared to the derived OATs, but also did not meet the obtained OATs for VPA or SPA. Only the "very poor/fair" fitness subgroup showed an MPA threshold, following the paradigm of the CTs (standard METs, and VO_2max_ intensity categories) comparable to the conventional one. In addition, higher OATs were derived using METs_60+_ (OATs_60+_) than those expressed in standard METs (OATs_standard_), regardless of the strategy followed to categorize the physical intensity (VO_2max_ or VTs). Therefore, those METs intensity thresholds based on standard METs should not be used when assuming a markedly lower 1 MET value, such as METs_60+_. Furthermore, profound differences were also observed when comparing OATs derived according to fitness status. Therefore, future studies classifying physical intensity in older adults should not avoid this critical issue. Alternatively, this study provides resources to the scientific community for the adaptation of the METs system in older adults, using both standard METs and METs_60+_, and according to the cardiorespiratory fitness of older adults. Furthermore, the use of OATs based on the VTs intensity categories is also encourage as an alternative to %VO_2max_ categories. Still, future studies should be performed to understand the actual influence of using these alternative OATs compared to CTs from a clinical and epidemiological perspective.

### Supplementary Information

Supplementary Material 1.

## Data Availability

The datasets generated and/or analysed during the current study are not publicly available due to institutional restrictions but can be obtained from the corresponding author upon reasonable request.

## Abbreviations

ACSM: American College of Sports Medicine
ACSMTs: METs intensity thresholds for the older population by the ACSM
BMI: Body mass index
CTs: Conventional METs intensity thresholds
GXT: Graded maximal exercise test
HR: Heart rate
HPA: Heavy-intensity physical activity
LPA: Light-intensity physical activity
METs: Metabolic equivalents
METs_60+_: Multiples of the older adult-based RMR value of 2.7 mL O_2_·kg^−^^1^·min^−^^1^
MPA: Moderate-intensity physical activity
OATs: METs intensity thresholds from older adults ≥ 60 years old
OATs_standard_: OATs expressed in standard METs
OATs_60+_: OATs expressed in METs_60+_
RMR: Resting metabolic rate
SPA: Severe-intensity physical activity
VerT: Supramaximal constant load verification test
VO_2max_: Maximal aerobic capacity
VPA: Vigorous-intensity physical activity
VTs: Ventilatory thresholds
VT_1_: Ventilatory threshold 1
VT_2_: Ventilatory threshold 2
