# Supplementary material for: Metabolic equivalents intensity thresholds for physical activity classification in older adults
Source: Eur Rev Aging Phys Act. 2024 May 21;21:14. doi: 10.1186/s11556-024-00348-5 (PMC11110193; doi:10.1186/s11556-024-00348-5)
Supplement: Supplementary file 1 — Supplementary Material 1. [file 11556_2024_348_MOESM1_ESM.docx]

**Title:** Metabolic Equivalents Thresholds for Physical Activity Intensity Classification in Older Adults.

| **Protocol** | **Trained individuals** | **Sedentary individuals** |
| --- | --- | --- |
| **GXT** | Stationary resting period:  Duration: 5 min  Pedaling cadence: 0 rpm | Stationary resting period:  Duration: 5 min  Pedaling cadence: 0 rpm |
|  | Warm-up period:  Duration: 5 min  Load: Males: 30W  Females: 15W  Pedaling cadence: 50-70 rpm | Warm-up period:  Duration: 5 min  Load: Males: 15W  Females: 10W  Pedaling cadence: 50-70 rpm |
|  | Load period:  Duration: until exhaustion  Load: Males: 4W/12s  Females: 3W/12s  Pedaling cadence: 60-90 rpm | Load period:  Duration: until exhaustion  Load: Males: 3W/12s  Females: 2W/12s  Pedaling cadence: 60-90 rpm |
|  | Active recovery period:  Duration: 2 min  Pedaling cadence: Ad libitum | Active recovery period:  Duration: 2 min  Pedaling cadence: Ad libitum |
|  | Passive recovery period:  Duration: 10 min  Pedaling cadence: 0 rpm | Passive recovery period:  Duration: 10 min  Pedaling cadence: 0 rpm |
| **VerT** | Total duration: until exhaustion  Warm-up period:  Duration: 30 s  Load: 0 W  Pedaling cadence: 60-90 rpm | Total duration: until exhaustion  Warm-up period:  Duration: 30 s  Load: 0 W  Pedaling cadence: 60-90 rpm |
|  | Submaximal period:  Duration: 1 min  Load: 50% W_max_  Pedaling cadence: 60-90 rpm | Submaximal period:  Duration: 1 min  Load: 50% W_max_  Pedaling cadence: 60-90 rpm |
|  | Supramaximal period:  Duration: until exhaustion  Load: 110% W_max_  Pedaling cadence: 60-90 rpm | Supramaximal period:  Duration: until exhaustion  Load: 110% W_max_  Pedaling cadence: 60-90 rpm |
|  | Active recovery period:  Duration: Undetermined  Pedaling cadence: Ad libitum | Active recovery period:  Duration: Undetermined  Pedaling cadence: Ad libitum |

**Supplementary table 1.** GXT and VerT protocols (FenotipAGING study)

GXT graded exercise test, VerT verification test.

| **Protocol** | **Men** | **Women** |
| --- | --- | --- |
| **GXT** | Warm-up resting (unloaded):  Duration: 3 min  Load: 0W  Pedaling cadence: 40-50 rpm | Warm-up resting (unloaded):  Duration: 3 min  Load: 0W  Pedaling cadence: 40-50 rpm |
|  | Warm-up period:  Duration: 3 min  Load: 15W  Pedaling cadence: 60-90 rpm | Warm-up period:  Duration: 3 min  Load: 10W  Pedaling cadence: 60-90 rpm |
|  | Load period:  Duration: until exhaustion  Load: 1W/5s  Pedaling cadence: 60-90 rpm | Load period:  Duration: until exhaustion  Load: 1W/7s  Pedaling cadence: 60-90 rpm |
|  | Active recovery period:  Duration: 3 min  Load: 10W  Pedaling cadence: Ad libitum | Active recovery period:  Duration: 3 min  Load: 10W  Pedaling cadence: Ad libitum |
|  | Passive recovery period:  Duration: 10 min  Pedaling cadence: 0 rpm | Passive recovery period:  Duration: 10 min  Pedaling cadence: 0 rpm |
| **VerT** | Total duration: until exhaustion  Warm-up period:  Duration: 30 s  Load: 0 W  Pedaling cadence: 60-90 rpm | Total duration: until exhaustion  Warm-up period:  Duration: 30 s  Load: 0 W  Pedaling cadence: 60-90 rpm |
|  | Submaximal period:  Duration: 1 min  Load: 50% W_max_  Pedaling cadence: 60-90 rpm | Submaximal period:  Duration: 1 min  Load: 50% W_max_  Pedaling cadence: 60-90 rpm |
|  | Supramaximal period:  Duration: until exhaustion  Load: 110% W_max_  Pedaling cadence: 60-90 rpm | Supramaximal period:  Duration: until exhaustion  Load: 110% W_max_  Pedaling cadence: 60-90 rpm |
|  | Active recovery period:  Duration: Undetermined  Pedaling cadence: Ad libitum | Active recovery period:  Duration: Undetermined  Pedaling cadence: Ad libitum |

**Supplementary table 2.** GXT and VerT protocols (PRO-Training study)

GXT graded exercise test, VerT verification test.

**
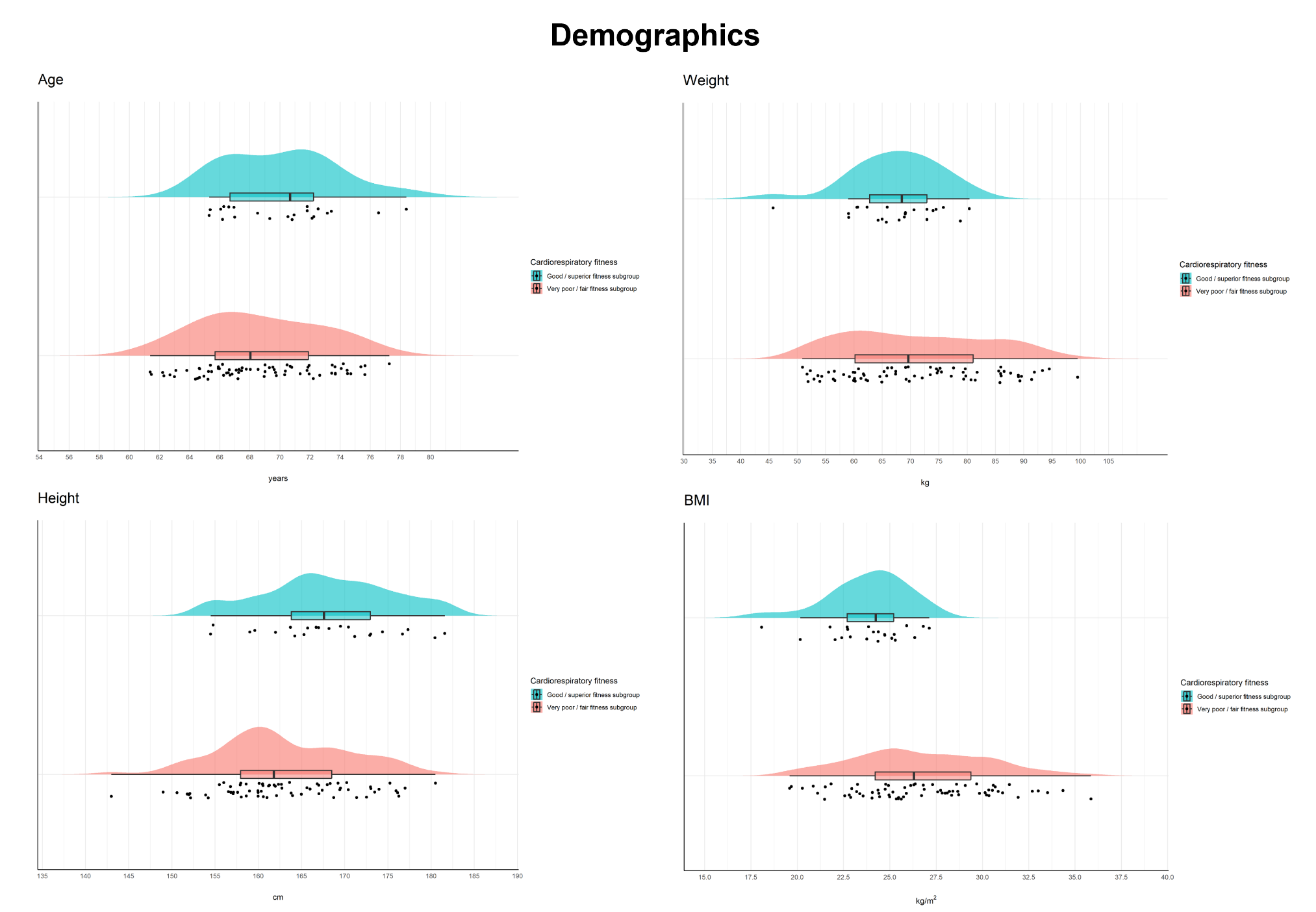
**

**Supplementary figure 1.** Raincloud plot of demographic variables by cardiorespiratory fitness subgroup.

**
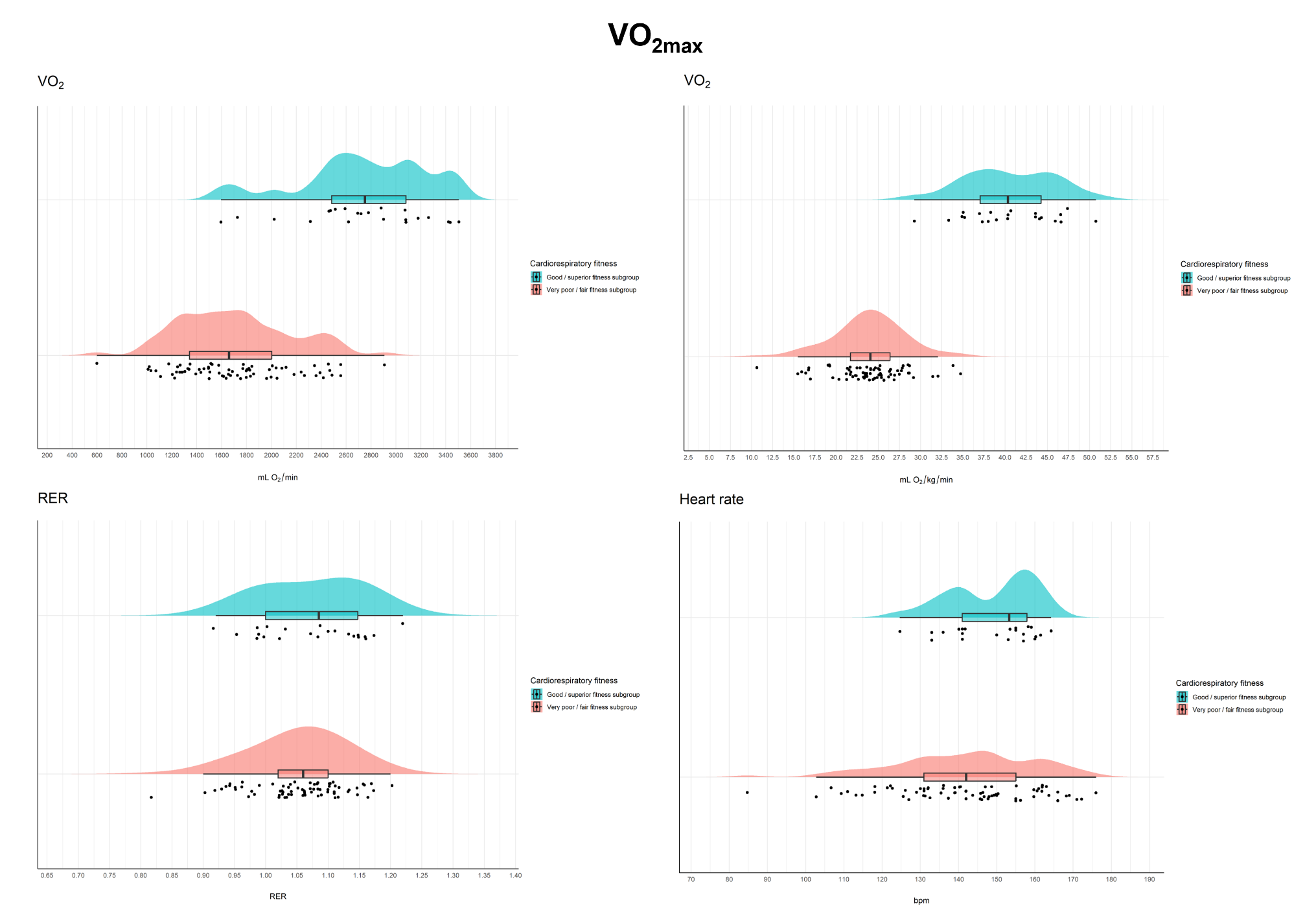
**

**Supplementary figure 2.** Raincloud plot of VO_2max_ variables by cardiorespiratory fitness subgroup.

**
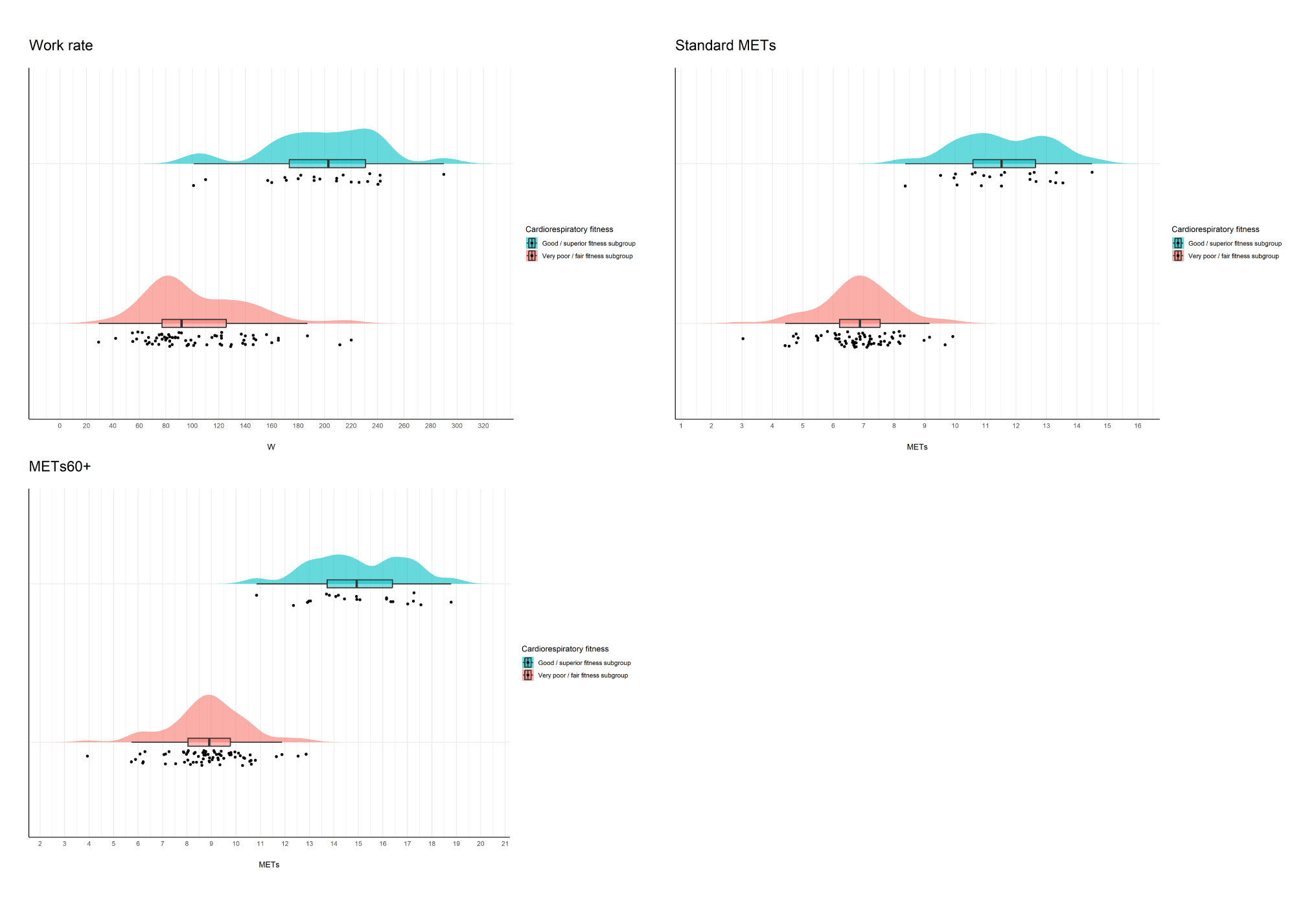
**

**Supplementary figure 2.** Raincloud plot of VO_2max_ variables by cardiorespiratory fitness subgroup (continued).

**
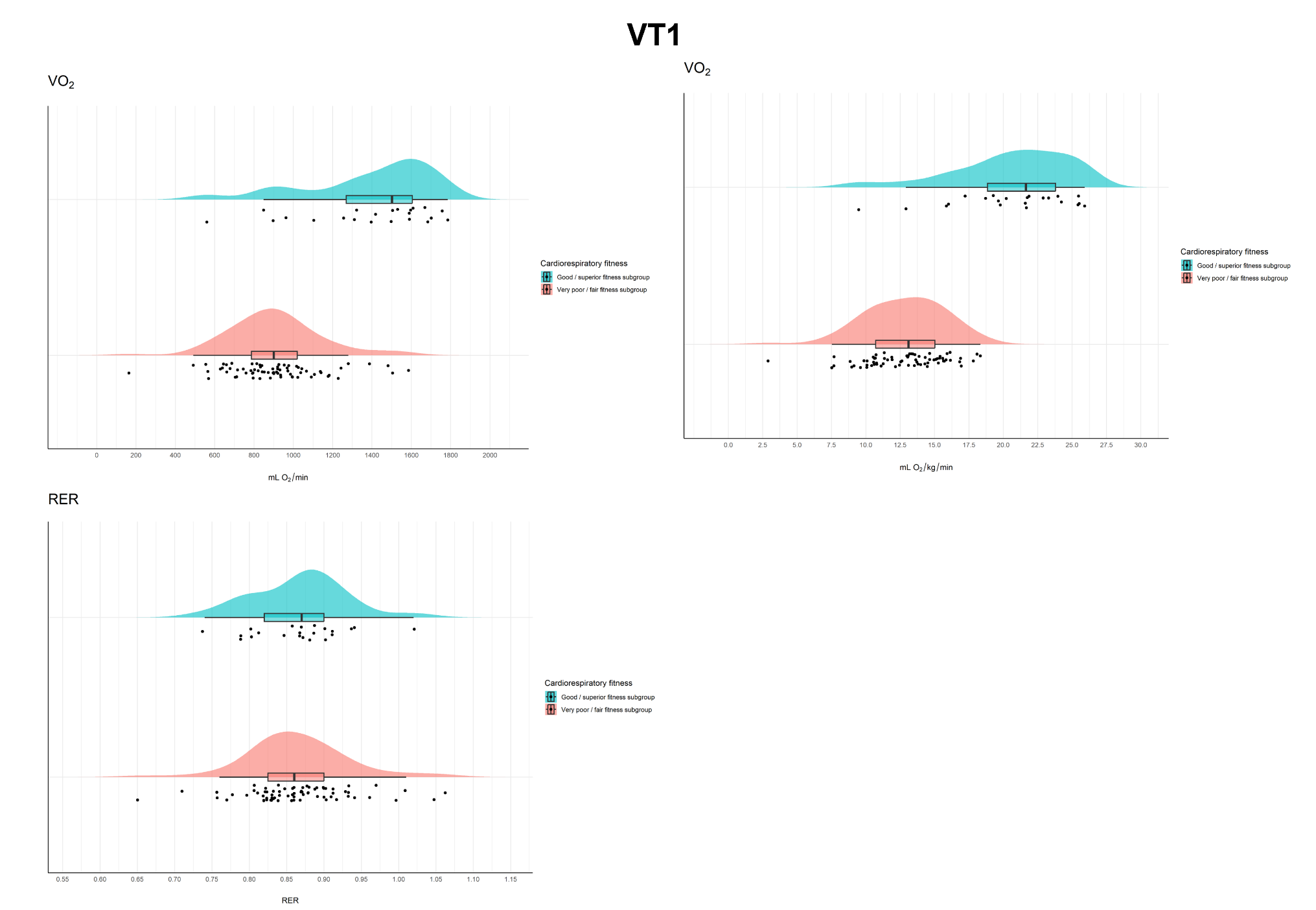
**

**Supplementary figure 3.** Raincloud plot of VT_1_ variables by cardiorespiratory fitness subgroup.

**
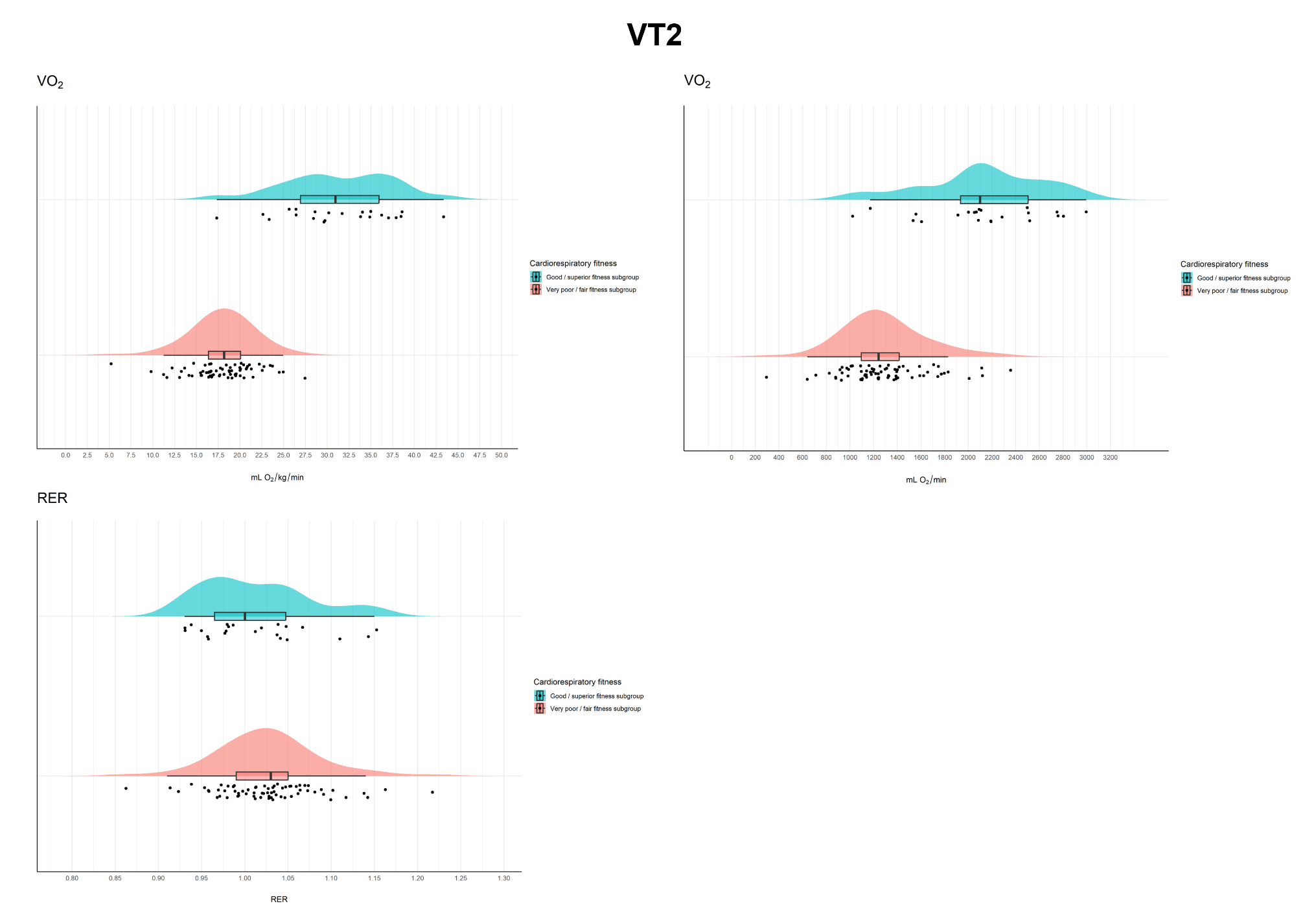
**

**Supplementary figure 4.** Raincloud plot of VT_2_ variables by cardiorespiratory fitness subgroup.

**
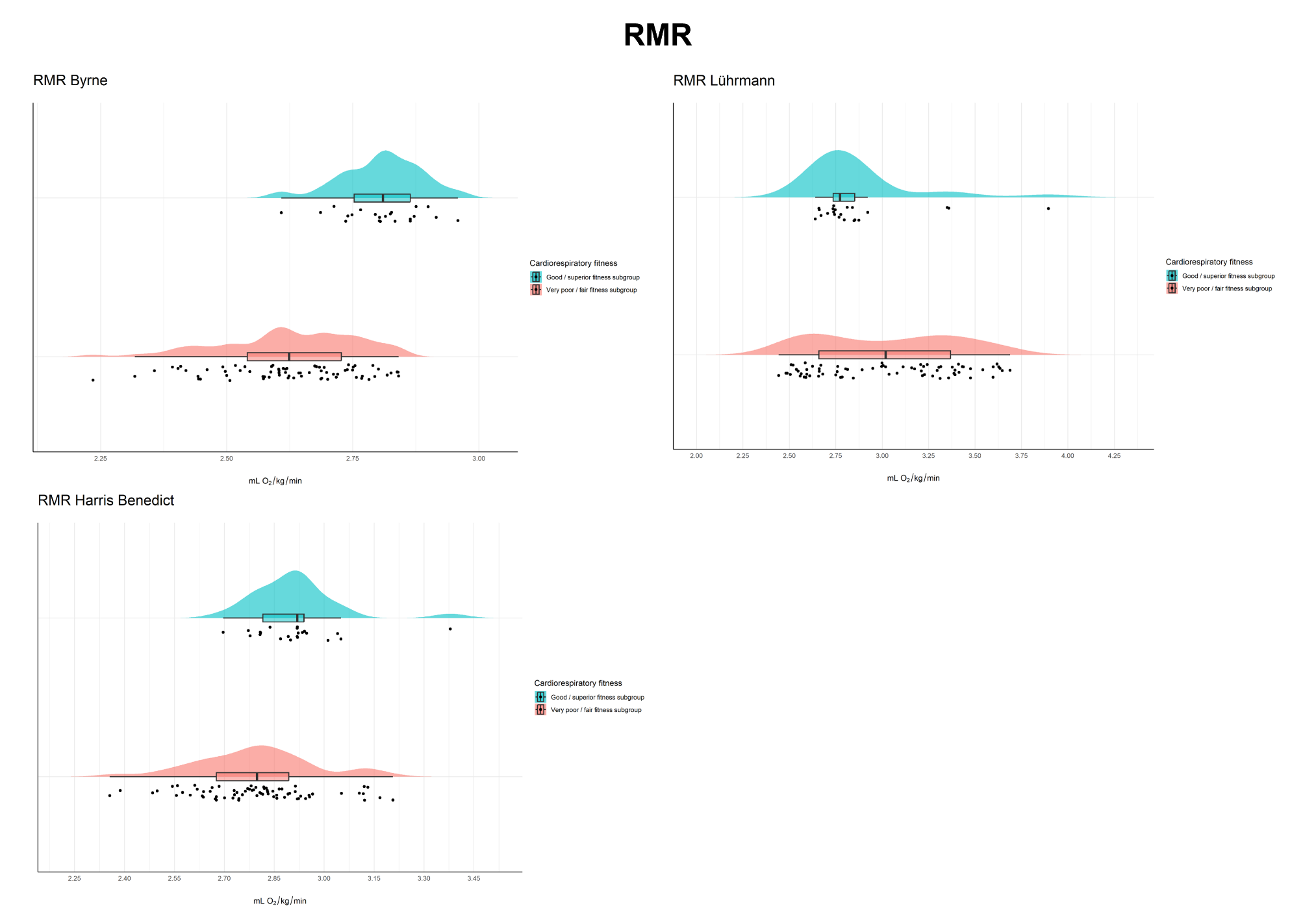
**

**Supplementary figure 5.** Raincloud plot of estimated RMR variables by cardiorespiratory fitness subgroup.


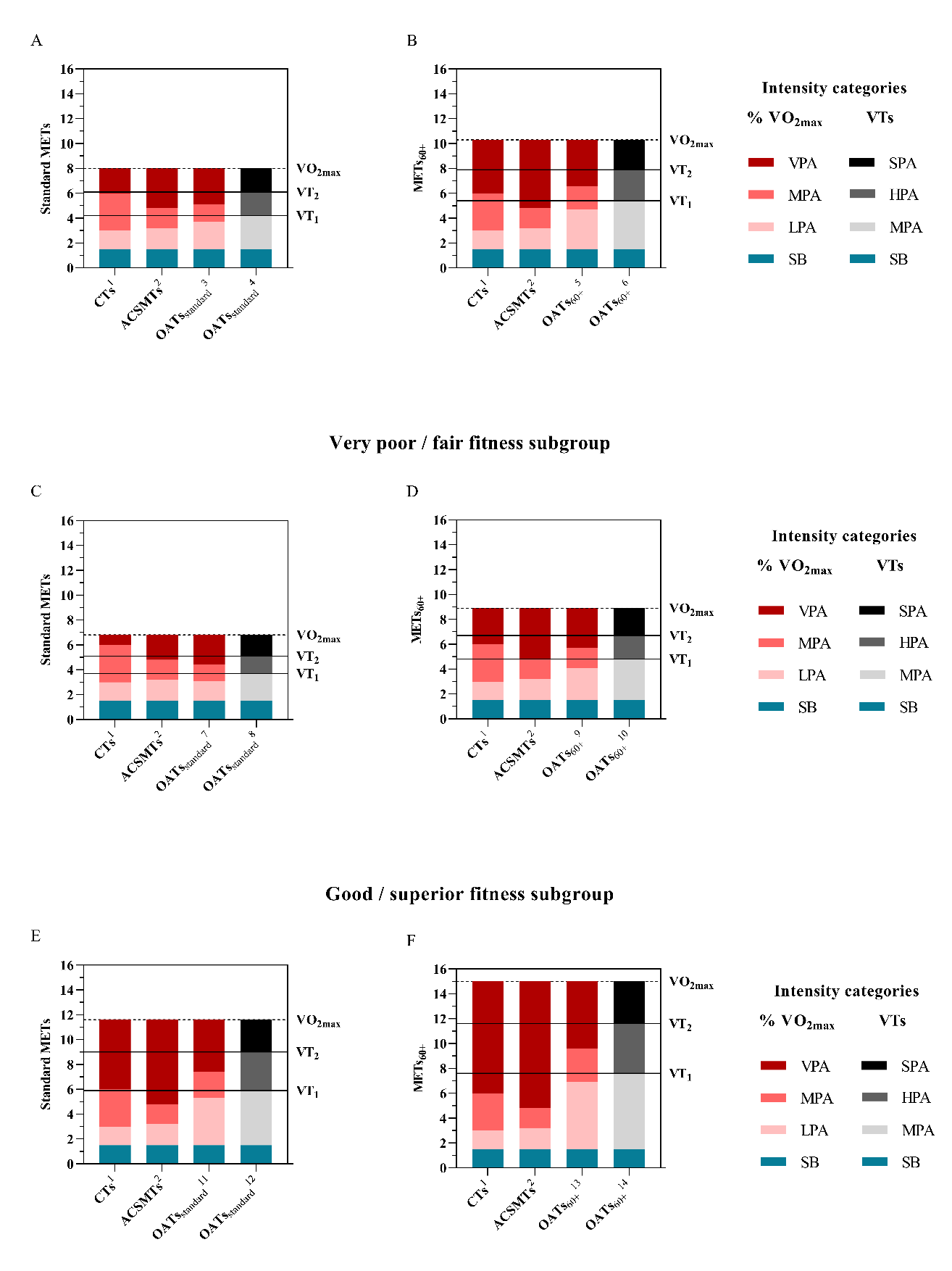


**Supplementary figure 6.** Overall and fitness-specific sample OATs ranges compared to the ranges of the CTs and the ACSMTs.

ACSM: American College of Sports Medicine, ACSMTs: METs intensity thresholds by the ACSM for older adults, CTs: conventional METs intensity thresholds, HPA: heavy-intensity physical activity, LPA: light-intensity physical activity, METs: metabolic equivalents, MPA: moderate-intensity physical activity, OATs: METs intensity thresholds for older adults ≥ 60 years old, OATs_standard_: METs intensity thresholds expressed in standard METs (VO_2_/3.5 mL O_2_·kg^-1^·min^-1^), OATs_60+_: METs intensity thresholds expressed in METs_60+_ (VO_2_/2.7 mL O_2_·kg^-1^·min^-1^), SB: sedentary behaviour, SPA: severe-intensity physical activity, VO_2max_: maximal oxygen uptake, VPA: vigorous-intensity physical activity, VTs: ventilatory thresholds, VT_1_: ventilatory threshold 1, VT_2_: ventilatory threshold 2. ^1^[SB] ≤ 1.5 METs; [LPA] > 1.5 to < 3.0 METs; [MPA] ≥ 3.0 to < 6.0 METs; [VPA] ≥ 6.0 METs, ^2^[SB] ≤ 1.5 METs; [LPA] > 1.5 to < 3.2 METs; [MPA] ≥ 3.2 to < 4.8 METs; [VPA] ≥ 4.8 METs, ^3^[SB] ≤ 1.5 METs, [LPA] > 1.5 to < 3.7 METs, [MPA] ≥ 3.7 to < 5.1 METs, [VPA] ≥ 5.1 METs; ^4^[SB] ≤ 1.5 METs; [MPA] > 1.5 to < 4.2 METs, [HPA] ≥ 4.2 to < 6.1 METs, [SPA] ≥ 6.1 METs; ^5^[SB] ≤ 1.5 METs, [LPA] > 1.5 to < 4.7 METs, [MPA] ≥ 4.7 to < 6.6 METs, [VPA] ≥ 6.6 METs; ^6^[SB] ≤ 1.5 METs, [MPA] > 1.5 to < 5.4 METs, [HPA] ≥ 5.4 to < 7.9 METs, [SPA] ≥ 7.9 METs, ^7^[SB] ≤ 1.5 METs; [LPA] > 1.5 to < 3.1 METs; [MPA] ≥ 3.1 to < 4.4 METs; [VPA] ≥ 4.4 METs, ^8^[SB] ≤ 1.5 METs; [MPA] > 1.5 to < 3.7 METs; [HPA] ≥ 3.7 to < 5.1 METs; [SPA] ≥ 5.1 METs, ^9^[SB] ≤ 1.5 METs; [LPA] > 1.5 to < 4.1 METs; [MPA] ≥ 4.1 to < 5.7 METs; [VPA] ≥ 5.7 METs, ^10^[SB] ≤ 1.5 METs; [MPA] > 1.5 to < 4.8 METs; [HPA] ≥ 4.8 to < 6.7 METs; [SPA] ≥ 6.7 METs, ^11^[SB] ≤ 1.5 METs; [LPA] > 1.5 to < 5.3 METs; [MPA] ≥ 5.3 to < 7.4 METs; [VPA] ≥ 7.4 METs, ^12^[SB] ≤ 1.5 METs; [MPA] > 1.5 to < 5.9 METs; [HPA] ≥ 5.9 to < 9.0 METs; [SPA] ≥ 9.0 METs, ^13^[SB] ≤ 1.5 METs; [LPA] > 1.5 to < 6.9 METs; [MPA] ≥ 6.9 to < 9.6 METs; [VPA] ≥ 9.6 METs, ^14^[SB] ≤ 1.5 METs; [MPA] > 1.5 to < 7.6 METs; [HPA] ≥ 7.6 to < 11.6 METs; [SPA] ≥ 11.6 METs.
